# BODE index: A predictor of hospitalization and severity in chronic obstructive pulmonary disease patients

**DOI:** 10.6026/973206300211831

**Published:** 2025-07-31

**Authors:** Dasari Ajay, Mini Bhatnagar, Namilla Chandra

**Affiliations:** 1 Department of Medicine, MM Institute of Medical Sciences & Research, Mullana, Ambala, Haryana, India; 2 Department of radiation oncology, Mehdi Nawaj Jung college Osmania University, Hyderabad, Telangana, India

**Keywords:** BODE Index, chronic obstructive lung disease (COPD), GOLD Grade, hospitalization, spirometry

## Abstract

The BODE index (Body Mass Index, Airflow Obstruction, Dyspnoea, Exercise tolerance) as a predictor of hospitalization and disease severity among Chronic obstructive lung disease (COPD) patients is of interest. Each patient's BODE index was calculated for 120 cases classified by the GOLD criteria. A strong correlation was found between the BODE index, GOLD grades and hospitalization frequency. ROC analysis confirmed that the BODE index significantly predicts severe COPD, with an AUC of 0.716. A cut-off value of >3 showed moderate sensitivity (62.1%) and specificity (62.6%) for predicting hospital admissions. Thus, the BODE index is a reliable tool for evaluating COPD severity and hospitalization risk, aligning closely with GOLD spirometry grades.

## Background:

Chronic Obstructive Pulmonary Disease (COPD) is a syndrome characterized by chronic airflow obstruction. COPD can include emphysema, chronic bronchitis, and small airway disease in varying combinations [1]. About 9-10% of persons aged > 40 worldwide have physiologically characterised chronic obstructive pulmonary disease (GOLD stage 2 or greater). COPD is the top cause of morbidity and mortality worldwide and anticipated to become the 3rd largest cause [2]. COPD is a prominent source of morbidity worldwide, especially in underdeveloped nations. COPD, an obstructive and progressive respiratory illness, is also linked to decreased physical activity and psychological issues, which increase disability and poor health-related quality of life (HRQoL) [3]. The WHO forecasts it will be the third leading cause of death by 2030 [4]. The main sign of airflow restriction in COPD is a decrease in FEV1. One physiological characteristic, forced expiratory volume in one second is used to categorise COPD patients' death risk. It measures lungs' air capacity. The Forced Expiratory Volume (FEV1) measures lung airflow. The FEV1 decreases in COPD patients due to airway constriction or inflammation. COPD is diagnosed when FEV1 drops to FVC. Calculate the FEV1 /FVC ratio to see if the drop is disproportionate. A FEV1/FVC ratio of <0.70 indicates COPD diagnosis [5]. Pathological alterations include excess extracellular matrix deposition, airway wall thickness, and mucus hypersecretions that constrict airways. Structure changes caused by inflammatory cytokines, proteinases, and oxidative stress chemicals can block airways. COPD patients often have weight loss, muscle wasting, hypoproteinaemia, and tissue depletion, as well as breathing difficulties, sputum production, and wheezing [6]. COPD patients are prone to heart disease, lung cancer, and other illnesses. COPD is most often caused by emphysema and chronic bronchitis. The main cause of COPD is smoking. People exposed to fuel fumes from cooking and heating in poorly ventilated homes typically gets it. The BODE index-Body mass index, airflow obstruction, dyspnoea, and exercise-is a multidimensional scoring system and capacity indicator used to examine COPD patients and predict long-term outcomes [7]. Severity in COPD includes four independent predictors of survival: BMI, FEV1, functional dyspnoea, and 6MWD exercise capacity [8]. The multidimensional classification system that predicts COPD mortality has emerged in recent decades due to greater understanding of COPD's systemic nature. Nowadays, BODE index predicts COPD mortality and severity better than FEV1. Global initiative for Chronic Obstructive Lung Disease (GOLD) says BODE index predicts mortality from any cause and respiratory causes better than FEV1-based staging [9]. Low-grade chronic systemic inflammation is now regarded as a key part of COPD. Inflammatory cytokines have been extensively studied in COPD's natural history. In addition to inflammation, proteases and antiproteases and oxidants and antioxidants (oxidative stress) in the lungs contribute to COPD. This condition burdens individuals and government health-care programmes since the expense of treatment and evaluation is directly proportional to the pulmonary and extra pulmonary components [10]. The process of providing treatment to the most needed patients from deficient resources can be extremely difficult in diseases which affect a large proportion of patients. BODE index is utilised for this in COPD patients [11]. Therefore, it is of interest to evaluate the efficacy of BODE index as a predictor of hospitalization and severity of the disease in patients with COPD and to correlate BODE index with spirometry.

## Materials and Methods:

A prospective observational study was conducted at the Department of Internal Medicine, M.M. Institute of Medical Sciences, Mullana, Ambala, involving 120 COPD patients who provided informed consent. Consecutive sampling was used and certain cases were excluded, including those with bronchial asthma, inability to perform spirometry or the six-minute walk test, life-threatening illnesses, acute COPD exacerbations and chronic liver or kidney diseases.

COPD diagnosis followed the GOLD criteria, which included:

[1] A persistent cough with sputum production for at least three months in two consecutive years.

[2] Exertional dyspnoea.

[3] Physical examination revealing airflow obstruction signs, such as prolonged expiration and expiratory wheezing, along with hyperinflation indicators.

[4] Spirometry showing a post-bronchodilator FEV1/FVC ratio below 0.70.

Each patient's smoking history and personal and family medical histories, was recorded. Height and weight were measured, with BMI calculated using the formula: BMI = Weight (kg) / Height^2^ (m). Spirometry was performed at enrolment and 20 minutes post-salbutamol nebulization, following American Thoracic Society standards. The FEV1 and FVC values were calculated using established prediction equations, adjusted for age, height and sex. The procedure was repeated to ensure accuracy, with the average result used. Dyspnoea severity was assessed using the Modified Medical Research Council (MMRC) dyspnoea scale, ranging from Grade 0 (no dyspnoea) to Grade 4 (dyspnoea during minimal activities). The six-minute walk test was done twice, with a 30-minute rest in between and the average distance covered was recorded. Patients were instructed to walk as far as possible within six minutes, including any rest breaks. The BODE index, which includes BMI, FEV1, six-minute walk test distance and MMRC dyspnoea score, was calculated for each patient. Points were assigned from 0 (minimum) to 3 (maximum) for each parameter:

[1] BMI: 0 for BMI >21, 1 for BMI ≤21

[2] FEV1: 0 for ≥65%, 1 for 50-64%, 2 for 36-49% and 3 for ≤35%

[3] Six-minute walk test: 0 for >350 meters, 1 for 150-249 meters and 3 for <150 meters

[4] MMRC dyspnoea: 0 for class 0, 1 for class I, 2 for class II and 3 for class III.

The total score ranged from 0 to 10, with the following classification:

[1] 0-2 for mild COPD

[2] 3-5 for moderate COPD

[3] 6 or higher for severe COPD.

## Statistical analysis:

Data were recorded on a pre-designed proforma. Qualitative data were presented as frequencies and percentages, with associations assessed using the Chi-Square test. Quantitative data were expressed as Mean ± SD and group comparisons were made using the unpaired t-test for normally distributed data or the Mann-Whitney test for non-normally distributed data. Statistical significance was defined as a p-value < 0.05. The optimal cut-off and screening efficacy of the BODE index were evaluated using Receiver Operating Characteristic (ROC) curve analysis, with SPSS Version 26.0 employed for statistical analysis.

## Results:

The average age of participants was 61.53 years, with 58.3% in the elderly group (over 60 years). The cohort had a male predominance (70% males, 30% females), yielding a male-to-female ratio of 2.33:1. A majority, 61.7%, were current smokers, while 32.5% had quit after being diagnosed with COPD and 5.8% were non-smokers. The mean duration of COPD was 9.73 years, with 45% of cases lasting more than 10 years. According to the GOLD criteria, 50% of participants exhibited mild symptoms, 36.7% had moderate symptoms and 13.3% had severe symptoms. Based on the BODE index, 56.7% were in the mild category, 30% in the moderate category and 13.3% in the severe category. A history of hospitalization was reported by 24.2% of cases, with a mortality rate of 0.8% (1 case) (Table 1 - see PDF). A significant association was found between the BODE index and GOLD grading, with agreement in 88.2% of mild cases, 81.8% of moderate cases and 100% of severe cases (p<0.01) (Table 2 - see PDF, Figure 1 - see PDF). The mean BODE index was 3.66 for patients requiring hospitalization, compared to 1.55 for those not requiring hospitalization (p<0.01). Hospitalization rates were 16.2% in the mild BODE group, 19.4% in the moderate group and 68.8% in the severe group (p<0.01) (Figure 2 - see PDF). At a BODE index cut-off of >2, sensitivity was 69% and specificity was 56%. At a cut-off of >3, sensitivity was 62.1% and specificity 62.6%. At >4, sensitivity was 44.8% and specificity 92.3%.

## Discussion:

Chronic Obstructive Pulmonary Disease (COPD) is a prominent contributor to illness and disability on a global scale, especially in less developed nations. FEV1 is very important for diagnosis and assessing severity of the disease. FEV1 is a good marker of disease progression and mortality but FEV1 does not correlate well with the degree of dyspnoea [12], and the change in FEV1 does not reflect the rate of decline in patients' health [13]. COPD is a multisystem disorder FEV1 alone cannot determine the outcome in these patients. BODE Index is a relatively simple approach to identifying disease severity using a combination predicted FEV1 %, Six-minute walk test, mMMRC dyspnoea scale and body mass index. It is a better predictor of subsequent survival than any component singly [14]. In present study, we aimed to evaluate the efficacy of BODE index as a predictor of hospitalization and severity of the disease in patients with chronic obstructive pulmonary disease (COPD). A substantial correlation was found between the BODE index and GOLD grading in present study. Similar association was also observed for hospitalization. The ROC analysis revealed that the BODE index was determined to be a significant predictor of severe COPD patients. Pavani *et al.* [15] in another similar study observed hospital stay as 1 day, 7.13 days and 12.05 days in mild, moderate and severe BODE index categories respectively (p<0.01). Sarkar *et al.* [16] (2015) studied the correlations between the BODE index and GOLD classification of COPD severity with health-related quality of life (HRQoL). Very strong correlations were found between BODE quartiles and HRQoL scores (P = 0.914; P < 0.01). In contrast, GOLD classes showed moderate correlation with total HRQoL scores (P = 0.590; P < 0.01). Ong *et al.* [7] hypothesized that the BODE index would better predict hospitalization for COPD than FEV1 alone. Using Poisson regression analysis, a significant effect of BODE score on the number of hospital admissions was found (incidence rate ratio, 1.20; 95% confidence interval [CI], 1.15 to 1.25; p < 0.001). In comparison, there was a significant but smaller effect of the FEV1 percentage of predicted on the number of hospital admissions (incidence rate ratio, 0.08; 95% CI, 0.04 to 0.16; p < 0.001). Ko *et al.* [17] study also observed that baseline BODE index was predictive of both the survival and readmissions to hospital for AECOPD by Cox regression analysis (p < 0.001 for both survival and readmissions). To summarize, current study found that the BODE index is a reliable predictor of hospitalisation and disease severity in patients with chronic obstructive pulmonary disease (COPD). The BODE Index demonstrated a strong correlation with spirometry measurements of GOLD grade. A BODE Index score greater than 3 demonstrated a sensitivity of 61.1% and specificity of 62.6% in predicting hospitalisation in individuals with chronic obstructive pulmonary disease. Moberg *et al.* [18] study also observed similar findings in a large cohort of 674 COPD patients undergoing pulmonary rehabilitation, where 84.9% had FEV_1_ ≤ 50% predicted, and 48.2% died over a mean follow-up of 66 months. The i-BODE index was reported to be 5.72 (SD = 1.86), and 62.5% had at least one COPD-related hospitalization during follow-up.

## Limitations of this study:

[1] The study sample was relatively small and the required sample size to detect significant differences in the predictive power between the BODE index and FEV1 was not pre-determined.

[2] Being a hospital-based study, the results may not be fully representative of the broader population.

[3] Caution is needed when generalizing these findings to populations outside of India, as regional variations in COPD manifestations have not been systematically compared.

[4] As a cross-sectional study, it is limited in its ability to determine if improvements in the BODE index lead to changes in the assessed parameters over time.

[5] Other potential factors, including the effects of medications, were not fully accounted for, which may have influenced the results.

## Conclusion:

BODE index (Body Mass Index, Airflow Obstruction, Dyspnoea and Exercise Tolerance) is a reliable predictor of hospitalization and disease severity in COPD. It is strongly correlated with spirometry-based GOLD grading for assessing disease severity. A BODE index score above 3 showed a sensitivity of 61.1% and specificity of 62.6% for predicting hospitalization in COPD patients.
